# Reproductive health for refugees by refugees in Guinea IV: Peer education and HIV knowledge, attitudes, and reported practices

**DOI:** 10.1186/1752-1505-5-10

**Published:** 2011-07-01

**Authors:** Aniek Woodward, Natasha Howard, Yaya Souare, Sarah Kollie, Anna von Roenne, Matthias Borchert

**Affiliations:** 1 London School of Hygiene & Tropical Medicine (LSHTM), Dept. of Disease Control, Keppel Street, London WC1E 7HT, UK; 2 Reproductive Health Group (RHG), Guéckédou, Guinea; 3 Deutsche Gesellschaft für Internationale Zusammenarbeit (GIZ) GmbH, Reichpietschufer 20, 10785 Berlin, Germany; 4 Institute of Tropical Medicine Antwerp, Nationalestraat 155, 2000 Antwerpen, Belgium; 5 Institute of Tropical Medicine and International Health (ITMIH), Charité-Universitätsmedizin Spandauer Damm 130, D-14050 Berlin, Germany

**Keywords:** refugees, health education, gender, chronic emergencies, HIV

## Abstract

**Background:**

Both conflict and HIV affect sub-Saharan Africa, and supportive approaches for HIV prevention among refugees are crucial. Peer education has been associated with improved HIV outcomes, though relatively little research has been published on refugee settings. The primary objective of this study was to assess whether exposure to refugee peer education was associated with improved HIV knowledge, attitudes, or practice outcomes among refugees in Guinea. Secondary objectives were to assess whether gender, age, or formal education were more strongly associated than peer education with improved HIV outcomes.

**Methods:**

Data was collected by cross-sectional survey from 889 reproductive-age men and women in 23 camps in the Forest Region of Guinea. Selected exposures (i.e. peer education, gender, formal education, age) were analysed for associations with HIV outcomes using logistic regression odds ratios (OR).

**Results:**

Most participants (88%) had heard of HIV, particularly those exposed to peer or formal education. Most correctly identified ways to protect themselves, while maintaining misconceptions about HIV transmission. Women and those exposed to either peer or formal education had significantly fewer misconceptions. Half of participants considered themselves at risk of HIV, women with 52% higher odds than men (adjusted OR 1.52, 95%CI 1.01-2.29). Participants exposed to peer education had more than twice the odds of reporting having made HIV-avoidant behavioural changes than unexposed participants (72% versus 58%; adjusted OR 2.49, 95%CI 1.52-4.08). While women had 57% lower odds than men of reporting HIV-avoidant behavioural changes (OR 0.43, 95%CI 0.31-0.60), women exposed to peer education had greater odds than exposed men of reporting HIV-avoidant changes (OR 2.70 versus OR 1.95). Staying faithful (66%) was the most frequent behavioural change reported.

**Conclusions:**

Peer education was most strongly associated with reported HIV-avoidant behaviour change. Gender was most associated with HIV knowledge and risk perception. Refugee women had fewer misconceptions than men had, but were more likely to report HIV risk and less likely to report making behavioural changes. Peer education appears promising for HIV interventions in chronic-emergency settings, if gender disparities and related barriers to condom usage are also addressed.

## Background

Both conflict and the human immunodeficiency virus (HIV) markedly affect sub-Saharan Africa [1]. In 2009, 43.3 million people were forcibly displaced worldwide including 3.1 million refugees in sub-Saharan Africa [2]. Of approximately 33.4 million people living with HIV (PLHIV) worldwide, 67% are in sub-Saharan Africa [2-4]. It might seem logical that displaced populations are at increased HIV risk, due to disrupted social structures and health services, increased sexual violence, poverty and deprivation [5-10]. However, research suggests HIV prevalence is no higher in refugee populations [1,11-13]. Several complex factors appear to determine how HIV affects refugees, including pre-crisis HIV prevalence in refugee and host populations, interaction between refugees and host populations, camp health and information services, and exposure to violence [1,5,14].

Despite decreasing HIV incidence, prevalence is rising as PLHIV live longer [15]. HIV prevention, treatment, care and support are now essential components of overall protection for refugees in post-crisis settings [16]. Improvements in availability of antiretroviral therapies in low-income countries has not translated into access for many refugees, supporting the continued importance of prevention efforts [17-19]. The decades since the policy shift of the International Conference on Population and Development (Cairo, 1994) and the Fourth World Conference on Women (Beijing, 1995) have seen an explosion of guidelines and policies on sexual and reproductive health (SRH) in crisis settings, including HIV prevention and antiretroviral therapy [20-22]. Relief efforts emphasise acute-phase mortality reduction, through Sphere guidelines, the minimum initial service package (MISP), and specific resources on refugees and HIV [23-25]. However, effective implementation is challenging and HIV indicators among refugees in post-acute and chronic humanitarian emergency settings are often poor [5,8]. Global and regional estimates of refugee PLHIV were first published in 2008, suggesting a lack of accurate indicators [8].

Peer education interventions have been associated with improved HIV knowledge, attitudes, and practices (e.g. increased condom use) in developing countries [26-29]. Peer-education research has focused on school settings and high-risk groups, and its effectiveness in refugee settings is still unclear. Tanaka et al (2008), the only publication found on refugee-led HIV education in chronic emergencies, showed a reduction in HIV risk behaviours among Congolese refugees in Tanzania [5].

### Study setting

From 1989 to 2004, conflicts in Liberia and Sierra Leone displaced over 500,000 people into the Forest Region of neighbouring Guinea [1,30]. Civil war in Sierra Leone lasted until 2002, and major refugee influxes in both early and late 1990s challenged Guinean health services [30]. The estimated HIV prevalence in adults of reproductive age (15-49 years) in Guinea rose from 0.2 to 2.2% and the number of PLHIV from 5,000 to 81,000 between 1990 and 2007, indicating a need for HIV prevention and related health promotion in Guinea [31].

The United Nations High Commissioner for Refugees (UNHCR) arranged for refugees to receive free treatment from Guinean facilities. However, many refugees expressed dissatisfaction with government SRH services [32]. In 1995, a group of refugee midwives and laywomen received funding and technical support from GTZ to organise the '*Reproductive Health Group' *(RHG). Somewhat unusually for the humanitarian field, RHG was a local, refugee-led, non-governmental organisation. RHG aimed to improve services for refugees in Guéckédou and Kissidougou prefectures. Von Roenne et al provide a detailed description of the RHG/GTZ 'reproductive health for refugees by refugees' model [32]. RHG seconded refugee nurses and midwives to Guinean health facilities and trained refugee laywomen to provide reproductive health education, referrals, and contraceptives for the refugee community. RHG drama groups accessed those refugees considered less likely to contact health services (e.g. men, young people) [32,33].

### Objectives

This paper is the fourth in a series evaluating the 'reproductive health for refugees by refugees' model [33-35]. It analyses data from a 1999 cross-sectional, questionnaire-based interview survey among refugees in areas supported by RHG for the previous four years. The primary objective was to assess whether exposure to refugee-led health education (i.e. peer education) was associated with improved HIV knowledge, attitudes, or practices. Secondary objectives were to assess whether gender, age, or formal education were more strongly associated with HIV knowledge, attitudes, or practices than was peer education and to discuss whether findings might be applicable to other chronic emergency settings.

## Methods

### Study design and data collection

Additional methodological details are published in Howard et al [36]. The target population was reproductive-age (15 to 49) male and female refugees from an estimated population of 250,000 living in 48 camps across the Forest Region of Guinea. First, 45 clusters of households were selected randomly from 23 camps, with probability of selection proportional to camp size. Second, a stratified sample of ten men and ten women per cluster was selected randomly from household lists. Weighting was not used, as there were equal numbers of men and women in the total refugee population. Sample size was calculated to detect a difference of 10% versus 20% between strata of equal size with 80% power and 95% confidence interval (95%CI), accounting for clustering. Participation was voluntary, informed, and not remunerated. Ethical approval was provided by the Ministry of Public Health in Guinea and the London School of Hygiene & Tropical Medicine in the UK.

The questionnaire, adapted from those used in similar low-income settings, was conducted in English and piloted outside the study area. Interviewers were recruited from the refugee community, trained and supervised, and the same sex as participants. The questionnaire used 'AIDS' instead of 'HIV' as participants were more familiar with this term. Data was double-entered in Epi-Info™6 using standard range and consistency checks [36].

### Data analysis

Data was analysed using Stata^®^11.0, to determine associations between selected exposures and HIV outcomes. Odds ratios (ORs) were calculated using logistic regression to adjust for confounding.

Peer education was categorised as *exposed *if participants reported RHG facilitators or drama groups as their main source of sexually-transmitted infection (STI) information and *unexposed *if not. Participants who had not heard of STIs were included in the latter group, as STI and HIV information was provided concurrently by RHG. Gender was coded to compare *women *to *men*. Age compared *youth *(15-24 years) to *mature *(25-49 years) adults. Education compared *education *(attended any formal schooling) to *no education *(attended no formal schooling).

Potential confounders, based on the literature and chi-square association tests, included gender, youth, education, ever having been married, age at sexual debut (defined as first penetrative sexual intercourse) and peer education for secondary analysis. All confounders, except sexual debut (coded categorically), were binary to increase cell sizes and improve power. Confounders were retained in multivariate logistic regression models if they changed ORs by at least 10%, after accounting for clustering using robust standard errors methods.

## Results

### Demographics

Response rates exceeded 95% and the final sample analysed was 889 participants, 445 men and 444 women. Table 1 shows demographic variables stratified by gender. Women had three times higher odds than men of having no formal education (OR 2.97, adjusted for ever having married; 95%CI 2.16-4.07) and over four times higher odds of being young (OR 4.26, adjusted for education, ever married; 95%CI 2.69-6.74). More than half of participants arrived in camps after 1996, most (97%) from Sierra Leone. Most participants reported themselves as Protestant (40%) or Muslim (41%). Sexual debut was above age 15 for most (87%) participants. Women had over six times higher odds than men of ever having married (OR 6.43, adjusted for education, age; 95%CI 3.87-10.68) and forty times higher odds of having married before age 18 (74% versus 6%; OR 40.19, adjusted for education, sexual debut; 95%CI 23.1-70.0). Of ever-married participants, women were significantly more likely to be currently widowed/divorced (OR 1.80; 95%CI 1.02-3.17) or living separately from their spouse (OR 2.47, adjusted for education; 95%CI 1.59-3.87).

**Table 1:** Demographic characteristics, comparing women to men

Demographic variables	Men (%)	Women (%)	OR (95%CI)^1^
*All participants:*	*n = 445 (100)*	*n = 444 (100)*	

Age**			
Youth (15-24)	162 (36)	190 (43)	
Mature (25-49)	283 (64)	254 (57)	4.26^b,d ^(2.69-6.74)
***Country of origin***			
Sierra Leone	436 (98)	432 (97)	0.76 (0.34-1.68)
Liberia	7 (2)	12 (3)	1.65 (0.73-3.71)
Other^+^	2 (0)	0 (0)	-
***Arrival in camp***			
Before 1996	202 (45)	188 (42)	
1996 or later	243 (55)	256 (58)	1.08^b-d ^(0.91-1.28)
Education**			
No formal education	181 (41)	316 (71)	
Some formal education	264 (59)	128 (29)	2.97^a,b,d ^(2.16-4.07)
Marital status**			
Ever married	275 (62)	375 (84)	
Never married	170 (38)	69 (16)	6.43^c,d ^(3.87-10.68)
***Religion***			
Catholic	82 (18)	88 (20)	1.27^b,c,d ^(0.93-1.75)
Protestant	173 (39)	184 (41)	1.08^a,c,d ^(0.85-1.38)
Muslim*	190 (43)	172 (39)	0.72^b,c,d ^(0.55-0.93)

*Sexually experienced participants:*	*n = 392 (100)*	*n = 418 (100)*	

***Age at sexual debut***			
14 years or less	40 (10)	62 (15)	
15 years or older/Unknown	352 (90)	356 (85)	1.56^a,b ^(0.94-2.57)

*Ever married participants:*	*n = 275 (100)*	*n = 375 (100)*	

Age at marriage^++,^**			
<18 years old	16 (6)	277 (74)	
≥ 18 years old	259 (94)	97 (26)	40.19^b,c ^(23.1-70.0)
Marital status*			
Widowed/Separated	24 (8)	55 (12)	
Currently married	251 (91)	320 (85)	1.80^a-d ^(1.02-3.17)
Residence of spouse**			
Living separately	38 (14)	100 (27)	
Living together in camp	237 (86)	275 (73)	2.47^b-d ^(1.59-3.87)

NB: *Χ^2 ^p-value ≤ 0.05; **Χ^2 ^p-value ≤ 0.001. ^+^OR calculation only relevant and displayed if cell n ≥ 5. ^++^One participant dropped because she did not give her age at marriage. ¹Adjusted for education, age, ever married, and age at sexual debut unless outcome is adjusted variable. ^a ^Not adjusted for education; ^b ^Not adjusted for age; ^c ^Not adjusted for ever married; ^d ^Not adjusted for age at sexual debut.

### Peer education

Table 2 shows associations between exposure to peer education and HIV knowledge, attitudes, and practices. The majority (88%) had heard of HIV, with exposed participants having over twice the odds of unexposed participants of having heard of HIV (OR 2.19; 95%CI 1.58-3.05). HIV knowledge was measured by eight true/false questions on prevention. Commonest accurate responses were staying with one faithful partner (95%), using clean needles (93%), and using condoms during sex (92%). Commonest incorrect responses were avoiding insect bites (69%), avoiding public toilets (50%), avoiding sharing food with (41%) or touching PLHIV (37%), and eating healthy food (36%). Exposed participants were consistently more likely to respond correctly. The five questions for which this difference was significant were staying faithful (OR 3.24, adjusted for gender; 95%CI 1.62-6.44), condom use (OR 1.91; 95%CI 1.15-3.16), avoiding public toilets (OR 1.70; 95%CI 1.22-2.38), eating healthily (OR 1.55; 95%CI 1.09-2.20), and sharing food with PLHIV (OR 1.52; 95%CI 1.10-2.10).

**Table 2:** HIV knowledge, attitudes and practices, comparing those exposed to RHG health education to those unexposed

Variables	Unexposed (%)	Exposed (%)	OR (95%CI)^1^
**2a) Knowledge**			

*All participants:*	*n = 380 (100)*	*n = 509 (100)*	

Heard of HIV**	316 (83)	466 (92)	
Never heard of HIV	64 (17)	43 (8)	2.19^a-e ^(1.58-3.05)

*All who heard about HIV:*	*n = 316 (100)*	*n = 466 (100)*	

Correctly answered the following statements:			
People cannot protect themselves from HIV by having good food*	186 (59)	321 (69)	1.55^a-e ^(1.09-2.20)
People can protect themselves from HIV by staying with one faithful partner**	293 (93)	456 (98)	3.24^a-d ^(1.62-6.44)
People cannot protect themselves from HIV by avoiding public toilets*	134 (43)	260 (56)	1.70^a-e ^(1.22-2.38)
People can protect themselves from HIV by using condoms during sex*	284 (90)	440 (94)	1.91^a-e ^(1.15-3.16)
People cannot protect themselves from HIV by avoiding touching a person who has HIV	187 (59)	308 (66)	1.34^a-e ^(0.98-1.85)
People cannot protect themselves from HIV by avoiding sharing food with person who has HIV*	171 (54)	299 (64)	1.52^a-e ^(1.10-2.10)
People cannot protect themselves from HIV by avoiding being bitten by mosquitoes or similar insects	89 (28)	165 (35)	1.27^a-d ^(0.90-1.78)
People can protect themselves from HIV by making sure any injection they have is done with a clean needle	291 (92)	442 (95)	1.58^a-e ^(0.87-2.88)
Knows a relative, friend or colleague with HIV	15 (5)	27 (6)	
Doesn't know anyone with HIV/Not sure	300 (95)	439 (94)	1.15^a-e ^(0.59-2.24)

**2b) Attitudes**	**Unexposed (%)**	**Exposed (%)**	**OR (95%CI)^1^**

*All who've heard of HIV:*	*n = 316 (100)*	*N = 466 (100)*	

I think HIV exists^+^	312 (99)	460 (99)	-
A person infected with HIV can sometimes look healthy*	62 (20)	122 (26)	1.45^a-e ^(1.02-2.06)
A woman infected with HIV can give birth to a child infected with HIV	257 (82)	403 (86)	1.44^a-e ^(0.92-2.27)
There is some risk I could catch HIV	168 (53)	230 (49)	
There is no risk that I could catch HIV	148 (47)	236 (51)	0.86^a-e ^(0.58-1.28)

**2c) Practices**	**Unexposed (%)**	**Exposed (%)**	**OR (95%CI)^1^**

*All who've heard of HIV:*	*n = 316 (100)*	*N = 466 (100)*	

I have made changes in my sexual behaviour to avoid HIV**	184 (58)	335 (72)	
I have not made changes in my sexual behaviour to avoid HIV	132 (42)	131 (28)	2.49^a-c ^(1.52-4.08)

*All who made HIV-avoidant changes:*	*n = 184 (100)*	*N = 335 (100)*	

I started making these changes more than 12 months ago	140 (76)	249 (74)	0.74^a-d ^(0.42-1.31)
Sexual behaviour changes reported:			
I am staying faithful to one partner*	126 (68)	215 (64)	0.59^a-d ^(0.41-0.87)
I am having fewer sexual partners than previously*	17 (9)	42 (13)	1.73^a-d ^(1.05-2.85)
I use condoms with casual partners	16 (9)	27 (8)	1.24^a-d ^(0.66-2.31)
I am abstaining	13 (7)	29 (9)	1.44^a-c,e ^(0.66-3.17)
I always use condoms	12 (7)	22 (7)	1.38^a-d ^(0.61-3.10)

NB: *Χ^2 ^p-value ≤ 0.05; **Χ^2 ^p-value ≤ 0.001. ^+^OR calculation only relevant and displayed if cell n ≥ 5. ¹Adjusted for education, age, ever married, age at sexual debut, and gender unless outcome is adjusted variable. ^a ^Not adjusted for education; ^b ^Not adjusted for age; ^c ^Not adjusted for ever married; ^d ^Not adjusted for age at sexual debut; ^e ^Not adjusted for gender.

No participants reported themselves as living with HIV, and few participants (5%) knew a relative, friend, or colleague living with HIV. However, 51% of participants considered themselves at risk of HIV. Most participants (84%) recognised vertical transmission from mother to infant. Exposed participants reported PLHIV could look healthy significantly more often than did unexposed participants (26% versus 20%; OR 1.45; 95%CI 1.02-2.06).

Exposed participants had more than twice the odds of unexposed participants of reporting changes in sexual behaviours to avoid HIV (72% versus 58%; OR 2.49, adjusted for gender, sexual debut; 95%CI 1.52-4.08). Staying faithful (66%) was the most frequently reported HIV-avoidant behavioural change. Exposed participants less frequently reported staying faithful (OR 0.59, adjusted for gender; 95%CI 0.41-0.87) and more frequently reported having fewer sexual partners (OR 1.73, adjusted for gender; 95%CI 1.05-2.85) than unexposed participants. Most participants (75%) reported making these changes over twelve months previously.

### Gender

Table 3a shows that women generally had higher HIV knowledge levels than men had. Women were also more likely than men to be exposed to peer education (56% versus 44%; OR 1.74; 95%CI 1.34-2.25). However, women had better HIV knowledge, whether exposed or unexposed to peer education (e.g.71% exposed and 66% unexposed women versus 56% exposed and 45% unexposed men knew people cannot protect themselves from HIV by avoiding sharing food with PLHIV). Table 3b shows that significantly more women than men reported themselves at risk of HIV (56% versus 46%; OR 1.52; 95%CI 1.01-2.29) and that vertical transmission from mother to infant can occur (88% versus 81%; OR 1.93, adjusted for education; 95%CI 1.16-3.21).

**Table 3:** HIV knowledge, attitudes and practices, comparing women to men

Variables	Men (%)	Women (%)	OR (95%CI)^1^
**3a) Knowledge**			

*All participants:*	*n = 445 (100)*	*N = 444 (100)*	


Heard of HIV	390 (88)	392 (88)	
Never heard of HIV	55 (12)	52 (12)	1.55^c,d ^(0.95-2.54)

*All who've heard of HIV:*	*n = 390 (100)*	*N = 392 (100)*	

Correctly answered the following statements:			
People cannot protect themselves from HIV by having good food*	239 (61)	268 (68)	1.68^b-e ^(1.17-2.42)
People can protect themselves from HIV by staying with one faithful partner	366 (94)	383 (98)	2.34^d ^(0.90-6.07)
People cannot protect themselves from HIV by avoiding public toilets	187 (48)	207 (53)	1.34^b-e ^(0.93-1.93)
People can protect themselves from HIV by using condoms during sexual intercourse	357 (92)	367 (94)	1.48^b-d ^(0.75-2.92)
People cannot protect themselves from HIV by avoiding touching a person who has HIV*	224 (57)	271 (69)	1.94^b,d,e ^(1.27-2.96)
People cannot protect themselves from HIV by avoiding sharing food with person who has HIV**	199 (51)	171 (69)	2.46^b-e ^(1.57-3.86)
People cannot protect themselves from HIV by avoiding being bitten by mosquitoes or similar insects**	90 (23)	164 (42)	2.90^b,c,e ^(1.83-4.60)
People can protect themselves from HIV by making sure any injection they have is done with a clean needle	359 (92)	374 (95)	1.92^e ^(0.78-4.76)
Knows a relative, friend or colleague with HIV	17 (4)	26 (7)	
Doesn't know anyone with HIV/Not sure	373 (96)	366 (93)	1.68^c,e ^(0.83-3.38)

**3b) Attitudes**	**Men (%)**	**Women (%)**	**OR (95%CI)^1^**

*All who've heard of HIV:*	*n = 390 (100)*	*N = 392 (100)*	

I think HIV exists	385 (99)	387 (99)	0.66^a,b,d,e ^(0.14-3.15)
A person infected with HIV can sometimes look healthy	100 (26)	84 (21)	0.79^a-e ^(0.53-1.18)
An HIV-infected woman can give birth to a child infected with HIV*	317 (81)	343 (88)	1.93^b-e ^(1.16-3.21)
I think I have some risk of catching HIV*	178 (46)	220 (56)	
I think I have no risk of catching HIV	212 (54)	172 (44)	1.52^a-e ^(1.01-2.29)

**3c) Practices**	**Men (%)**	**Women (%)**	**OR (95%CI)^1^**

*All who've heard of HIV:*	*n = 390 (100)*	*N = 392 (100)*	

I have made changes in my sexual behaviour to avoid HIV**	274 (70)	245 (62)	
I have not made changes in my sexual behaviour to avoid HIV	116 (30)	147 (38)	0.43^a,b ^(0.31-0.60)

*All who made HIV-avoidant changes:*	*n = 274 (100)*	*N = 245 (100)*	

I started making these changes more than 12 months ago*	183 (67)	206 (84)	2.59^a,d,e ^(1.44-4.67)
Sexual behaviour changes reported:			
Staying faithful to one partner**	142 (52)	199 (81)	3.36^b,d ^(2.27-4.98)
Fewer sexual partners than previously*	42 (16)	16 (7)	0.50^b,d ^(0.26-0.96)
Using condoms with casual partners**	37 (13)	6 (2)	0.17^b,d ^(0.06-0.45)
Abstinence	22 (8)	20 (8)	1.42^a,b,d ^(0.80-2.52)
Always using condoms^+^	30 (11)	4 (2)	-

NB: *Χ^2 ^p-value ≤ 0.05; **Χ^2 ^p-value ≤ 0.001. ^+^OR calculation only relevant and displayed if cell n ≥ 5. ¹Adjusted for education, age, ever married, age at sexual debut, and RHG health education unless outcome is adjusted variable. ^a ^Not adjusted for education; ^b ^Not adjusted for age; ^c ^Not adjusted for ever married; ^d ^Not adjusted for age at sexual debut; ^e ^Not adjusted for RHG health education.

Table 3c shows that women had significantly lower odds of having made HIV-avoidant behaviour changes (OR 0.43, adjusted for peer education exposure, ever married, sexual debut; 95%CI 0.31-0.60). However, women exposed to peer education had nearly three times higher odds of HIV-avoidant behavioural changes than unexposed women (OR 2.70, adjusted for formal education, age, ever married, sexual debut; 95%CI 1.56-4.65), while exposed men had nearly twice the odds of HIV-avoidant changes compared to unexposed men (OR 1.95, adjusted for sexual debut; 95%CI 1.06-3.60). Odds of reporting 'staying faithful,' were over three times greater for women than men (81% versus 52%; OR 3.36, adjusted for peer education exposure, ever married, education; 95%CI 2.27-4.98). Women were less likely to report having fewer sexual partners (OR 0.50, adjusted for peer education exposure, ever married, education; 95%CI 0.26-0.96) or increased condom usage with casual partners (OR 0.17, adjusted for peer education exposure, ever married, formal education; 95%CI 0.06-0.45). Of those reporting behavioural changes, women were more likely than men were to have made changes over twelve months previously (OR 2.59, adjusted for age, ever married; 95%CI 1.44-4.67). Peer education exposure was not associated with timing of behaviour changes for men, but was for women. Exposed women who had made HIV-avoidant changes had significantly lower odds than unexposed women of having made changes over twelve months ago (81% versus 94%; OR 0.20; 95%CI 0.06-0.62).

### Formal education

Table 4 compares participants with some formal education to those with no formal education. Participants with some education had twice the odds of having heard of HIV than those without formal education (OR 2.13; 95%CI 1.51-3.00). The former were also somewhat more knowledgeable about HIV. Formally-educated participants significantly more frequently correctly stated that people cannot protect themselves from HIV by eating healthily (OR 1.25; 95%CI 1.04-1.49), avoiding touching (OR 1.34, adjusted for gender; 95%CI 1.11-1.61) or sharing food (OR 1.22, adjusted for gender; 95%CI 1.03-1.43) with PLHIV, or avoiding insect bites (OR 1.29, adjusted for gender; 95%CI 1.07-1.56).

**Table 4:** HIV knowledge, attitudes and practices, comparing participants with some formal education to those with no formal education

Variables	No education (%)	Education (%)	OR (95%CI)^1^
**4a) Knowledge**			

*All participants:*	*n = 497 (100)*	*n = 392(100)*	

Heard of HIV**	408 (82)	374 (95)	
Never heard of HIV	89 (18)	18 (5)	2.13^a-e ^(1.51-3.00)

*All who've heard of HIV:*	*n = 408 (100)*	*n = 374 (100)*	

Correctly answered the following statements:			
People cannot protect themselves from HIV by having good food*	245 (60)	262 (70)	1.25^a-e ^(1.04-1.49)
People can protect themselves from HIV by staying with one faithful partner	391 (96)	358 (96)	0.99^a-e ^(0.73-1.34)
People cannot protect themselves from HIV by avoiding public toilets	197 (48)	197 (53)	1.09^a-e ^(0.93-1.29)
People can protect themselves from HIV by using condoms during sexual intercourse	373 (91)	351 (94)	1.20^a-e ^(0.90-1.59)
People cannot protect themselves from HIV by avoiding touching a person who has HIV*	243 (60)	252 (67)	1.34^b-e ^(1.11-1.61)
People cannot protect themselves from HIV by avoiding sharing food with person who has HIV*	241 (59)	229 (61)	1.22^b-e ^(1.03-1.43)
People cannot protect themselves from HIV by avoiding being bitten by mosquitoes or similar insects*	126 (31)	128 (34)	1.29^b-e ^(1.07-1.56)
People can protect themselves from HIV by making sure any injection they have is done with a clean needle	380 (93)	353 (94)	1.11^a-e ^(0.83-1.50)
Knows a relative, friend or colleague with HIV	18 (4)	25 (7)	
Doesn't know anyone with HIV/Not sure	390 (96)	349 (93)	1.25^a-e ^(0.87-1.79)

**4b) Attitudes**	**No education (%)**	**Education (%)**	**OR (95%CI)^1^**

*All who've heard of HIV:*	*n = 408 (100)*	*n = 374 (100)*	

I think HIV exists	403 (99)	369 (99)	1.23^a,b,d,e ^(0.66-2.29)
A person infected with HIV can sometimes look healthy	88 (22)	96 (26)	1.12^a-e ^(0.94-1.33)
An HIV-infected woman can give birth to a child infected with HIV	338 (83)	322 (86)	1.14 (0.93-1.41)
I think I have some risk of catching HIV*	222 (54)	174 (47)	
I think I have no risk of catching HIV	186 (46)	198 (53)	0.86^a-e ^(0.76-0.98)

**4c) Practices**	**No education (%)**	**Education (%)**	**OR (95%CI)^1^**

*All who've heard of HIV:*	*n = 408 (100)*	*n = 374 (100)*	

I have made changes in my sexual behaviour to avoid HIV	269 (66)	250 (67)	
I have not made changes in my sexual behaviour to avoid HIV	139 (34)	124 (33)	0.96^b-e ^(0.85-1.09)

*All who made HIV-avoidant changes:*	*n = 269 (100)*	*n = 250 (100)*	

I started making these changes more than 12 months ago	217 (81)	172 (69)	0.82^b-e ^(0.67-1.01)
Sexual behaviour changes reported:			
Staying faithful to one partner*	203 (75)	138 (55)	0.75^b-e ^(0.60-0.94)
Fewer sexual partners than previously	28 (10)	31 (12)	0.77^a,b,d,e ^(0.57-1.05)
Using condoms with casual partners*	10 (4)	33 (13)	1.64^b-e ^(1.01-2.64)
Abstinence	17 (6)	25 (10)	1.28^a-e ^(0.95-1.74)
Always using condoms	11 (4)	23 (9)	1.23^b-e ^(0.83-1.82)

NB: *Χ^2 ^p-value ≤ 0.05; **Χ^2 ^p-value ≤ 0.001. ¹Adjusted for gender, age, ever married, age at sexual debut, and RHG health education unless outcome is adjusted variable. ^a ^Not adjusted for gender; ^b ^Not adjusted for age; ^c ^Not adjusted for ever married; ^d ^Not adjusted for age at sexual debut; ^e ^Not adjusted for RHG health education.

Formally-educated participants less frequently reported themselves at risk of HIV than did participants without formal education (OR 0.86; 95%CI 0.76-0.98). The former less frequently reported staying faithful (55% versus 75%; OR 0.75, adjusted for gender; 95%CI 0.60-0.94) and more frequently reported condom use with casual partners as HIV-avoidant behaviour changes (13% versus 4%; OR 1.64, adjusted for gender; 95%CI 1.01-2.64). However, numbers were small. No strongly significant associations with peer education were found.

### Age

Mature participants (over age 25) appeared to have slightly more HIV knowledge, though no significant differences were found after adjusting for confounders. Mature participants more frequently reported having made HIV-avoidant behavioural changes than did younger participants (73% versus 56%), though this difference was not significant. Mature participants reported making behavioural changes over twelve months previously significantly more frequently than did younger participants (OR 2.07, adjusted for gender, ever married; 95%CI 1.16-3.69). No strongly significant associations with peer education were found.

## Discussion

### Peer education

Both peer education and gender were strongly associated with particular HIV knowledge, attitude, or practice outcomes. Interestingly, most participants knew they could protect themselves from HIV by staying faithful and using condoms and clean needles, while maintaining misconceptions about transmission. Both peer education and formal education were significantly associated with HIV knowledge. Similar results were found in an accompanying paper on sexually transmitted infections, supporting Tanaka et al's findings that peer education was associated with improved awareness of HIV risk and prevention methods [5,34]. However, transmission misconceptions could increase fear or avoidance of routine practices, such as using public toilets, and more importantly of PLHIV (e.g. not touching them or sharing food). Misconceptions could also distract refugees from effective prevention methods, as research in Malawi indicates many HIV health messages were not or only partly believed by participants [37]. Some misconceptions could also foster a degree of fatalism - e.g. if any mosquito can transmit HIV, then condoms offer insufficient protection, so why bother using them?

Importantly, peer education was positively associated with reported HIV-avoidant behaviour changes. However, 'staying faithful,' the most commonly reported HIV-avoidant behaviour change in this study, is only effective if both partners practice it. RHG facilitators distributed free condoms, but did not always have enough to meet demand. Condom 3-packs were sold in local markets at an approximate cost of 200 Francs Guinéens (US$0.28 in 2009 constants). However, ever (23%) and current (11%) condom usage was low [32,36]. Research indicates cultural factors, including influence from social elites (e.g. religious leaders, traditional healers), can affect sexual behaviours, perceived side effects, trust, and gender disparities [5,37-39]. Thus, health promotion among refugees should continue to reduce perceived barriers to condom use.

### Other exposures

Interestingly, peer education exposure was more strongly associated with HIV-avoidant behaviour changes for women than for men (i.e. OR 2.70 versus OR 1.95). Women demonstrated greater HIV knowledge than did men, despite lower educational attainment. While equal numbers of men and women had heard of HIV (88%), women reported significantly fewer misconceptions. This could be because women had greater exposure to RHG and peer education or even that they were more open to health education messages. Women may have learned about HIV through antenatal clinics, as parity was associated with increased reproductive health knowledge in accompanying papers [35,36]. In contrast, Tanaka et al found female Congolese refugees demonstrated lower knowledge levels and higher-risk practices than male refugees [5]. This may have been because female refugees in Guinea attended health services more frequently than did their Congolese counterparts, allowing greater exposure to health education. However, as Tanaka et al did not appear to account for confounders, there could be other reasons. More research in other refugee populations might help determine whether noted differences were associated with greater exposure or greater openness to peer education among female versus male refugees.

Significantly more women than men reported themselves at risk of HIV in this study. Riskier behaviours among women included significantly lower mean ages at sexual debut and marriage, and less reported condom usage (9% versus 37%) or current condom usage (3% versus 19%) than men [32,36]. In contrast, Rowley et al found that among refugees in Tanzania, men were more involved in high-risk sex than women [40]. This is partly explained by differences in risk outcomes, as Rowley et al focussed on number of casual partners and transactional sex in the last twelve months [40]. Research shows women are at higher risk of HIV infection, with gender disparities and consequent risks potentially worsening during displacement [1,6,10,17,19,40-47]. Beliefs that women should be sexually passive could decrease the opportunities for displaced women to actively protect themselves from HIV [48]. Limited access to education, work, or money could make women refugees dependent on male partners or transactional sex, limiting their control over timing or circumstances of sex [19,48]. Additionally, if women experience sexual violence or abuse, condom negotiation is unlikely [19,44,48].

Findings in Uganda indicate that although condom use was important in reducing HIV incidence, fewer sexual partners appeared more important [49]. In most cultures, having multiple partners is more socially acceptable for men than for women [50]. Men were more likely than were women to report having fewer, or using condoms with, casual partners as their HIV-related behaviour changes. Family-planning research in this population indicated approximately 27% polygyny, which could be either a risk (if involving casual sex) or protective factor (if in a faithful polygynous marriage) [36].

Gender differences in risk perceptions could indicate male risk perceptions were either inaccurately low or had decreased due to HIV-avoidant behaviour changes. Higher risk perceptions among women could consequently be due to risky sexual behaviours by their partners or lower likelihood of having made sexual behaviour changes themselves [50]. It seems probable that greater risk perceptions among women highlight the relative challenges for women in this population to protect themselves from HIV - as it was men who decided condom usage, and how and with whom to have sex. Female condoms were not available in this population, and it is unknown whether their use would have been accepted. Increasing condom distribution would not solve gender disparities, though it seems reasonable that a male-targeted condom promotion campaign could increase usage. Findings support global policy recommendations on the need for gender-sensitive solutions.

Young participants (ages 15-24) had similar knowledge levels to mature participants, contradicting findings from the Millennium Development Report and suggesting that health services and RGH support may have been more youth-friendly than men-friendly. Alternatively, men may have chosen not to access health information while young people did. Male outreach was conducted by RHG facilitators, who were generally female, possibly creating a barrier to male participation.

### Limitations

Much has changed since 1999 when data was initially collected. Implementers are far more knowledgeable about HIV control in emergency settings and have a broader range of tools available. However, while most of these refugees have now left Guinea, health issues in the country have not improved significantly and findings remain relevant. For example, antiretroviral therapies were not available in Guinea until 2002 and coverage was still low (9-10%) in the most recent figures from 2006, while coverage has increased in sub-Saharan Africa from 14% in 2005 to 43% in 2008 [15,51]. No participants reported living with HIV. Underreporting is possible, both due to sensitivities and because people may not have wanted to know their status as treatment was not yet available.

A mixed-methods approach would have been preferable for this study. Unfortunately, additional research was cancelled due to security issues, preventing qualitative data collection. Cross-sectional studies determine association not causality. HIV prevalence and related behaviour were measured through self-report, less reliable than objective measurement and vulnerable to underreporting. HIV transmission via sexual intercourse was addressed, as this is the main mode of transmission in sub-Saharan Africa [4].

Categorisation of some versus no education did not consider educational quality or level as few participants had more than 3-4 years of education. Reporting and observer bias were minimised through surveyor training and questionnaire piloting. Chance was reduced through robust standard errors methods. Residual confounding is possible, as data was not collected on number of casual partners, transactional sex, sexual violence, drug use, socio-economic status, or other variables that could affect HIV-related choices [40].

## Conclusions

This study gave insight into the effectiveness of refugee-led HIV education within a chronic-emergency camp setting. Refugee peer education appears useful, as it was positively associated with HIV knowledge, attitudes to risk, and HIV-avoidant practices. This suggests other technical support agencies could utilise the GTZ/RHG 'reproductive health for refugees by refugees' model and consider gender disparities for health promotion to be effective.

## Competing interests

AvR and YS are current employees of GTZ, while MB has worked as a GTZ consultant.

## Authors' contributions

AW and NH analysed the data, drafted the paper, and gave final approval of the version for publication. YS and SK contributed to conception and design, acquisition of data, and reviewing the paper. AvR conceived the study, and contributed to design, data interpretation, and reviewing the paper. MB designed the study, contributed to acquisition, analysis and interpretation of data, and critical revision of the paper. All authors approved the version for publication.
